# The angiogenic growth of cities

**DOI:** 10.1098/rsif.2023.0657

**Published:** 2024-04-03

**Authors:** Isabella Capel-Timms, David Levinson, Bahman Lahoorpoor, Sara Bonetti, Gabriele Manoli

**Affiliations:** ^1^ Department of Civil, Environmental and Geomatic Engineering, University College London, London, UK; ^2^ School of Civil Engineering, University of Sydney, Sydney, Australia; ^3^ Laboratory of Catchment Hydrology and Geomorphology, École Polytechnique Fédérale de Lausanne, Sion, Switzerland; ^4^ Laboratory of Urban and Environmental Systems, École Polytechnique Fédérale de Lausanne, Lausanne, Switzerland

**Keywords:** urban growth, population dynamics, transport networks, reaction–diffusion

## Abstract

Describing the space–time evolution of urban population is a fundamental challenge in the science of cities, yet a complete theoretical treatment of the underlying dynamics is still missing. Here, we first reconstruct the evolution of London (UK) over 180 years and show that urban growth consists of an initial phase of diffusion-limited growth, followed by the development of the railway transport network and a consequential shift from central to suburban living. Such dynamics—which are analogous to angiogenesis in biological systems—can be described by a minimalist reaction–diffusion model coupled with economic constraints and an adaptive transport network. We then test the generality of our approach by reproducing the evolution of Sydney, Australia, from 1851 to 2011. We show that the rail system coevolves with urban population, displaying hierarchical characteristics that remain constant over time unless large-scale interventions are put in place to alter the modes of transport. These results demonstrate that transport schemes are first-order controls of long-term urbanization patterns and efforts aimed at creating more sustainable and healthier cities require careful consideration of population–transport feedbacks.

## 1. Introduction

As urban populations continue to grow [1], it is becoming increasingly important to improve our quantitative understanding of urbanization processes to inform the design of more sustainable infrastructure, services and activities [2–4]. A central tenet of urban science is the description of how cities and their multiple sub-systems evolve in time and space (e.g. [5,6]) but a complete theoretical understanding of the controlling factors and dynamics is still missing (e.g. [7]). This constrains the possibility of informing planning and decision making with plausible scenarios of long-term urban development [8]. Cities are not just the sum of their parts, but the result of a self-organizing process (e.g. [8–11]) involving myriad complex interactions across multiple scales and agents (e.g. individuals, businesses, policy makers) [7,12,13]. Hence, if we are to effectively anticipate and quantify city behaviour, for the sake of future planning and design but also to anticipate global land use change, we must recognize and quantify these symbioses and feedbacks.

As they grow, cities increase in complexity in a way that is not fully controlled by urban planning efforts (e.g. [14]). Existing knowledge suggests that urbanization is an emergent phenomenon resulting from the superposition of smooth and ‘unregulated’ growth with centralized, large-scale interventions acting as punctuated changes in time [11,15]. Such theories, inspired by the statistical physics of complex systems, reveal that the small-scale heterogeneity of urban characteristics may be surrogated by a small number of rules governing the macroscopic behaviour of the system. This is a challenging problem however, as illustrated by the variety and limitations of current urban systems modelling approaches [16]. Existing agent-based models (e.g. [8,12]) integrate a large number of detailed, complex interactions, but the level of dependency on fine-tuning and the risk of overfitting hinder the generalizability of their predictions [13,17], especially when the system undergoes unexpected perturbations. Traditional urban economic models (e.g. [18]), on the other hand, employ fewer parameters but their link to empirical data is generally weak [13] and they may be inadequate to address complex real-world problems. Additionally, more recent economic approaches either require highly detailed information to describe specific purposes (e.g. migration costs, amenities, market access etc. [19]), or neglect important urban features (e.g. transport costs do not account for the network topography [13]), and rely on the assumption that the system is in equilibrium, which is unrealistic for real cities [13]. Statistical approaches also exist, such as diffusion limited aggregation (e.g. [20,21]) and gravity-based models (e.g. [22]), but they are largely confined to modelling the sprawl of single population clusters and the element of randomness involved makes causal analysis problematic, especially when considering feedbacks with other urban sectors (e.g. transport).

Reaction–diffusion (RD) approaches—inspired by the early work on morphogenesis (e.g. [23])—provide an attractive alternative for modelling urban systems. Differential equations describing RD processes, such as the Fisher–Kolmogorov equation [24], have been successfully employed to study population dynamics (e.g. [25]), and widely applied and tested in various fields (e.g. tumour growth, stem growth [26–29]). However, these ideas are less often applied to urban systems, where long-term and high-resolution data for model testing are generally lacking. A few examples exist [30–33], but the formation of urban patterns generally results from ad hoc choices of the model parameters, rather than socio-economic constraints based on real-world quantities (e.g. population growth, rent/transport costs, network geometry, etc.), and simulations are generally not compared with observations at the relevant spatio-temporal scales. Clearly, there are many potential factors and interactions influencing the growth and shape of urban settlements, including social, economic and geographical conditions. Hence, the main challenge for such a reduced-complexity description of city behaviour is to identify a minimal set of parameters and governing mechanisms, or ‘first principles’ [34], that describe quantitatively the space–time evolution of cities.

Here, we first analyse the development of London (UK), over a period of 180 years (1831–2011) and provide a novel, theoretical description of intra-city urban growth by combining an RD model with concepts of urban economics and network science. As a first-order approximation, we focus on the distribution of resident population and the structure of the railway network because of their connections to urban mobility and livability, from infrastructure to pollution and mortality risk (e.g. [5,35,36]). To assess the generality of our approach, we then analyse the long-term evolution of Sydney, Australia, a coastal city characterized by different environmental and socio-economic conditions.

We show that urban population, after an initial phase of intense densification, coevolves with the growth of the transport network, generating an ‘order by fluctuations’ [37] that results from the superposition of stochastic changes in the system (i.e. the creation of new network nodes), a deterministic response to such changes (i.e. diffusion and redistribution of resident population), and exogenous sources and sinks (resulting from large-scale land use interventions, government policy, etc.). The stochastic component of the model (i.e. the fluctuations) encodes innovation and decision-making processes, but also our incomplete knowledge of the system on such long time scales [37]. The mathematical formulation is general, allowing it to describe the rich dynamics of urban systems. It can therefore be employed to test the long-term effects of policies and regulations on demography and mobility. Note, however, that the aim here is not to capture fine scale heterogeneities and the exact network topography, but to quantify how population distribution and network properties coevolve over long time scales. This knowledge is key to exploring plausible urbanization scenarios and fostering science-based urban planning (e.g. [13,38]) in a way that recognizes the complex interactions governing the long-term evolution of population and infrastructure [39]. This is particularly crucial during this era of accelerated urbanization and digitalization, as cities are generally treated as large machines or logistic systems that can be controlled by top-down interventions when, in reality, they behave like complex adaptive systems which evolve—to a certain extent—like living organisms [40]. Our results support this hypothesis of instructive similarities between urban and biological systems [41], prompting a constructive dialogue across disciplines.

## 2. Growth phases of a metropolis: the case of London

**Figure 1.**
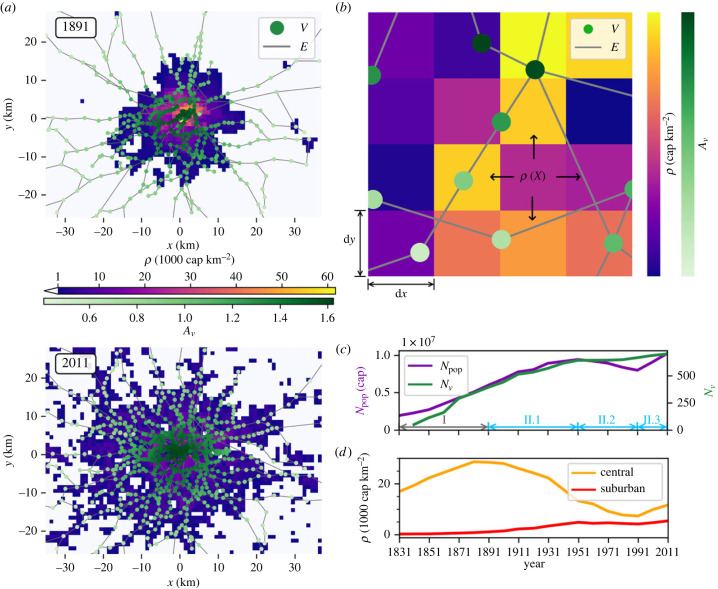
London space–time evolution. (*a*) Observed residential population density *ρ* ($1000\;{\mathrm{cap}\;\mathrm{km}}^{-2}$) and rail network (National Rail, Underground, Overground and DLR) for 1891 and 2011. Station nodes (*V*) are coloured by accessibility *A*_*v*_. Rail line edges (*E*, grey) are represented by straight lines, which connect stations according to the real network topography but are not representative of the actual geography. For the Greater London model domain, the spatial discretization is d*x* = d*y* = 1 km. (*b*) An illustration of the model interactions including the model grid, *ρ*, the diffusion of *ρ* (represented by arrows), the transport network station nodes (*V*) and rail line edges (*E*), are also shown. (*c*) Number of stations (*N*_*v*_, green) and total population (*N*_pop_, cap, purple) within the entire domain for the study period 1831–2011, and the phases of diffusion-limited (grey arrows) and reaction–diffusion (blue arrows) growth. (*d*) *ρ* (cap km^−2^) within representative central (orange) and suburban (red) areas.

London is often considered the poster child for modern metropolises and, in contrast to other cities (such as Paris [11]), its evolution was not influenced by large-scale planning until the 1940s (e.g. [42]). Data and studies show that the city reflects the typical evolution patterns of large metropolitan areas (figures 1*a*,*c*,*d*; figure SI.1 in the electronic supplementary material [42–44]), consisting of an initial phase of central densification followed by urban expansion in the suburbs (e.g. [45]) (figure 1*d*).

Before the introduction of the railway, the resident population of London was clustered around the centre of the city. Long-distance modes of transport were inefficient and reserved for the wealthy elite, so living close to the workplace was the most efficient solution for the majority of people [42]. After the introduction of the passenger rail network in 1836, the time–distance relation was drastically altered in the areas where it was present, allowing residents to move further out from the centre. However, the development of the rail network was then subject to competition between rail providers and restrictive policy, such as the exclusion of railways from the centre of the city [42] and, as such, it did not serve the majority of the population. Despite this, there is evidence of commuting occurring from 1851, when the City of London started to see a decline in resident population in favour of suburban development [42,46]. This is a typical pattern of intra-city migration, with people preferring lower density living as transport capabilities make the option more viable [47] (as illustrated also for Paris and Venice; figure SI.1 in the electronic supplementary material). The underground rail network was established in 1863, connecting mainline railway stations on the city edge with multiple stations in between. By 1891 (figure 1*a*), the rail network (including underground) had become fairly dense across the city, and suburban living became increasingly popular (figure 1*d*).

Suburban growth continued after this period, along with a steady increase in total population *N*_pop_ (cap). This increase was closely followed by growth in the number of stations (*N*_*v*_) across the domain (figure 1*c*). A post-war decline in *N*_pop_ then occurred, driven by decreases in the working population after industrial decline in the city centre [48] and government policy that encouraged the movement of the population [49]. During this period the suburban population stagnated (figure 1*d*), and the growth of the rail network slowed down. During the 1980s and 1990s, urban regeneration in the Docklands area established a new business district, attracting more people who sought increased economic activity to both the area itself and the wider city. *N*_*v*_ also increased (figure 1*c*), both as part of regeneration projects and in response to higher demand. These various stages in the growth of both London’s rail network and its population demonstrate periods of distinct changes in the space–time evolution of the city.

## 3. Mathematical model

### 3.1. Space–time dynamics of population density

To reproduce such urban development patterns, we can assume that population density *ρ*(*X*, *t*) is a continuous function of time *t* and space *X* = (*x*, *y*), defined over a two-dimensional domain $\Omega \in R^{2}$. In Ω, we define also an undirected graph *G* = (*V*, *E*), composed of a discrete number of vertices *V* (i.e. the railway stations) connected by edges *E* (i.e. the railway lines). Assuming that the time evolution of the transport network *G* is known, the observed dynamics of *ρ*(*X*, *t*) can be described by a RD equation (figure 1*b*):

3.1 $$\frac{\partial \rho }{\partial t}=D\left(\frac{\partial^{2}\rho }{\partial x^{2}}+\frac{\partial^{2}\rho }{\partial y^{2}}\right)+\eta (X,t)\cdot \xi (t)+R(X,t)-S(X,t),$$

where *ξ*(*t*) (cap km^−2^ yr^−1^) is the growth of the total population across the domain, which is distributed in space based on the local attractiveness factor *η* (itself a function of *ρ*(*X*, *t*) and *G*). $R(X,t)=R_{-}(X,t)+R_{+}(X,t)\;({\mathrm{cap}\;\mathrm{km}}^{-2}\;{\mathrm{yr}}^{-1})$ is an ‘internal’ redistribution of residents, accounting for a population sink (*R*_−_) that is redistributed across the domain (*R*_+_), with $\int_{\Omega }R(X,t)\;d\Omega =0$, while *S*(*X*, *t*) (cap km^−2^ yr^−1^) is an ‘external’ sink which removes population from the domain entirely, accounting for exogenous perturbations (here *S* ≠ 0 only in the period 1940–1990; see table 1 for details). The values and functional forms of these source/sink terms reflect different growth phases as explained in the next sub-sections. The population is allowed to diffuse throughout the domain Ω with diffusion coefficient *D* (km^2^ yr^−1^), which is assumed to be constant (in time and space) for the sake of simplicity. Note that while we are assuming that RD dynamics can be applied to urban systems, the idea of modelling urban development as a diffusion process is not new, as there is a large literature on the geographical diffusion of settlements (e.g. [32,50]) as well as urban sprawl and suburbanization [51]. Following Batty *et al.* [51], this approach ‘emphasizes local growth through diffusion [such that it] is characteristic of the way an urban fringe develops [···]. Urban areas grow around their edges primarily due the demand for new space which is translated through the usual process into new development’. Similarly, Bracken & Tuckwell [52] proposed a simple deterministic mathematical model for urban growth considering ‘local logistic growth, diffusion and an integral (killing) term representing the inhibitory effect of congested central areas on growth’. Yet this assumption is purely theoretical as, strictly speaking, there is no clear evidence that the ‘diffusion’ of urbanization is the result of random walks and mixing of population (e.g. [53]).

**Table 1.** Growth phases, their periods used within the London case study, and corresponding changes in the model equations.

name	description	domain period	equation details
I	early network (densification)	1831–1891	*Y* ∼ *ρ*, *R* = *S* = 0
II.1	suburbanization	1882–1951	*Y* ∼ (*C*_*L*_, *C*_*T*_), *R* ≠ 0, *S* = 0
II.2	population decline	1951–1991	*Y* ∼ (*C*_*L*_, *C*_*T*_), *R* ≠ 0, *S* ≠ 0
II.3	population regeneration	1992–2011	*Y* ∼ (*C*_*L*_, *C*_*T*_), *R* = 0, *S* = 0, *K* = *K*_II.3_

The term *ξ* represents exogenous, non-local, demographic processes describing the growth/decline of total domain population density, i.e. *ξ*(*t*) = d*P*(*t*)/d*t*, where $P(t)=a_{\Omega }^{-1}\int_{\Omega }\rho (X,t)\;d\Omega$ (cap km^−2^) and $a_{\Omega }$ is the model domain area (km^2^). *ξ* could involve, for example, resource supply dynamics (e.g. [3]), inter-urban migration with neighbouring cities, or Lévy flight (e.g. [6]). For this study *ξ* is assumed to follow a logistic formulation with carrying capacity *K* (cap km^−2^) and growth rate *r* (yr^−1^), in agreement with the observed London domain total population data (figure 1*c*) and population growth patterns observed in many cities across the world [3,54]:

3.2 $$\xi (t)=r\cdot P(t)\left(\frac{K-P(t)}{K}\right).$$

Equation (3.1) thus represents a non-local version of the Fisher–Kolmogorov equation which is a standard model for the space–time dynamics of a population of individuals that diffuse, grow, and compete for resources (e.g. [25]). The non-local term is employed here to link the vast literature on city-scale growth (e.g. [3,6]) with the observed spatial dynamics of population density. This formulation is general and relatively simple, thus facilitating future model applications and improvements—for example, alternative models of total population growth exist (e.g. [3,6,55]) and could be used to capture the growth phases of different cities.

#### 3.1.1. Influence of living and transport costs

The factor *η* distributes the new inhabitants across a heterogeneous landscape, accounting for differences in growth that occur due to interactions between *ρ* and the transport network. Following [5,34,56], *η* can be written as the Gibbs’ function of the net income *Y*(*X*, *t*), i.e.

3.3 $$\eta (X,t)=\frac{e^{\beta Y(X,t)}}{a_{\Omega }^{{\;}^{-}1}\int_{\Omega }e^{\beta Y(X,t)}\;d\Omega }.$$

The growth of the population and the transport network depends on living (*C*_*L*_(*X*, *t*)) and transport (*C*_*T*_(*X*, *t*)) costs, which are balanced against a constant gross income *Y*_gross_ to give a heterogeneous net income *Y*(*X*, *t*) following the work by Barthélemy & Flammini [5,57] (note that this a simplification for the sake of modelling purposes and ignores more detailed socio-economic processes, e.g. income segregation [58]). However, as observed for London and other cities (see figure 1*a*; figure SI.1 in the electronic supplementary material [42,59]), a simpler case can also exist when the network is less established (i.e. during periods of early densification), with *Y*(*X*, *t*) linked to the economic opportunity presented by a dense population rather than access to a station. Hence, we reformulate the net income *Y* as

$$\;\;\;\;\;Y(X,t)=\;\left\{ \begin{array}{cc} Y_{0}\rho {\left(X,t\right)}^{B}\;\; & \text{if}\;t<t_{I}\;(3.4a)\\ Y_{\mathrm{gross}}-C_{L}(X,t)-C_{T}(X,t) & \text{otherwise},\;(3.4b) \end{array}\right.$$

where *t*_*I*_ is the year defining the end of the densification phase (phase I, see also table 1), with base *per capita* income *Y*_0_ and densification exponent *B*. After this phase, the population responds to the growth of the rail network as travel times over long distances shorten and suburbanization starts to occur. *Y* becomes a balance between gross income *Y*_gross_ (constant for this study), *C*_*L*_ and *C*_*T*_. Living costs (accommodation) *C*_*L*_ are calculated considering the cumulative population in the local area:

3.5 $$C_{L}(X,t)=\kappa {\left(\int_{0}^{t}\rho (X,t)\;dt\right)}^{\tau },$$

where *κ* and *τ* are model parameters. We consider the cumulative population density rather than *ρ* (as done in [5]) because it is indicative of the existence of available buildings and the centrality associated with previous population growth, resulting in competition between office, retail, and residential land uses (e.g. bid-rent theory [60]). As such, *C*_*L*_ does not decrease with a sinking population, thus resolving the increase in rent/property values observed in most historical centres despite their decrease in residential population.

Transport costs *C*_*T*_(*X*, *t*) are estimated considering the network graph *G* = (*V*, *E*). The relative importance of an individual network node (*v*) within *G* is given by the node accessibility factor *A*_*v*_ (figure 1*b*). Various metrics can be used to quantify this, for example betweenness [5] or closeness centrality, or, for a more detailed case, the contribution of the network to the attainability of ‘opportunities’ such as jobs, activities or sales [61]. This model employs a simple approach, without loss of generality, by describing the interaction between each node and the rest of a network as the ratio of total travel distance between all other nodes and the network average travel distance, as detailed mathematically by Wang *et al.* [62] (see equations (SI.1)–(SI.3) in the electronic supplementary material). In this study, we use the inverse of the accessibility given by Wang *et al.*, such that more accessible stations have a higher value and therefore a lower transport cost. Stations with average accessibility have *A*_*v*_ = 1. To account for multiple nodes in an area, *C*_*T*_ is calculated using the *A*_*v*_ value of the node nearest to the grid cell *X* (figure 1*b*), and a non-local constant *h*. *C*_*T*_ is estimated following [5], but also considering the minimum distance to the nearest station, *d*_min_, to account for commuting to/from the railway, i.e.

3.6 $$C_{T}(X)=\mu d_{\min}(X)+\nu (h-\bar{A_{v}}(X)),$$

where $\bar{A_{v}}(X)$ is the average *A*_*v*_ when multiple nodes exist in the grid cell *X*. The term $h-\bar{A_{v}}(X)$ is guaranteed by *h* to be non-zero everywhere, while *μ* and *ν* are model parameters that regulate the balance between the costs to reach and use the transport network.

#### 3.1.2. Population redistribution and sink

Other population sources/sinks are considered here to reflect non-diffusive migration across the domain (*R*(*X*, *t*)) and removal of population (*S*(*X*, *t*)) during phases of population decline (e.g. as observed in figure 1*c*). We propose a simple approach that incorporates the aforementioned dynamics while retaining the economic constraints discussed earlier (equation 3.4*b*) [5]. This implies that areas with higher *Y* are more likely to retain population due to their attractiveness, and areas with lower *Y* might lose their population to either other areas of the domain or to outside of the domain. Specifically, internal redistribution is estimated based on the economic attractiveness of an area. Negative values of *Y* imply that costs are higher than the gross income, prompting residents to move to more convenient locations. Hence, the flux of residents leaving an area *X* is calculated as

$$\;R_{-}(X,t)=\left\{ \begin{array}{cc} r_{R}\cdot \rho {\left(X,t\right)}_{X,t-1} & \text{if}\;Y(X,t)<0\;\;\;(3.7a)\\ 0\;\; & \text{otherwise},\;\;\;\;(3.7b) \end{array}\right.$$

where *r*_*R*_ (yr^−1^) is the sink rate specific to *R*_−_.

To account for migration *within* the domain, *R*_−_ is then redistributed as

3.8 $$R_{+}(X,t)=\eta (X,t)\cdot a_{\Omega }^{-1}\int_{\Omega }R_{-}(X,t)\;d\Omega .$$

However, a migrating population can also leave the domain altogether due to exogenous factors acting on the city population, such as a post-war decline in population or the removal of industries. This is encoded in the sink term *S*(*X*, *t*), defined as

3.9 $$S(X,t)=r_{S}\cdot \rho (X,t)\cdot \eta_{S}(X,t),$$

where *r*_*S*_ (yr^−1^) is the sink rate for *S* and

3.10 $$\eta_{S}(X,t)=\frac{e^{-\beta_{S}Y(X,t)}}{a_{\Omega }^{-1}\int_{\Omega }\;e^{-\beta_{S}Y(X,t)}\;d\Omega }.$$

By formulating *η*_*S*_ in a similar way to equation (3.3), we assume that areas with lower *Y* experience a greater sink *S* as they are seen as less attractive, not only for emerging population but also for population potentially migrating.

#### 3.1.3. Phases of population growth

As discussed in §2, London has exhibited distinctive growth phases over the past two centuries. This is encoded in the model parameterization as illustrated in table 1. Specifically, a first phase (I) of localized diffusion-limited growth, is followed by a spatial spreading (II) influenced by the development of the rail network. For the case of London phase II is split into three parts (phases II.1, II.2 and II.3) which each describe different stages of RD growth.

City population is known to fluctuate through time, experiencing multiple periods of growth, decline and stagnation (e.g. [3]). Regrowth is also possible after decline as a city might seek to become more attractive, through, for example, urban regeneration projects [63], and start a new cycle of innovation and growth (e.g. [3]). An adaptive carrying capacity (maintaining the same growth rate *r*) can be used to reflect such punctuated dynamics [55]. For the sake of simplicity, we introduce here a new carrying capacity *K*_II.3_ to replace *K* in equation (3.2) when calculating *ξ* for the period 1992–2011 as estimated from population projections. More refined models (e.g. a delayed differential equation for the growth of the total population (e.g. [55,64])) could be considered in future works but are considered beyond the scope of this study.

Note that, in reality, the periods of these phases are not so distinct, i.e. changes have been more gradual than the implied sudden transitions which are necessary model simplifications. Additionally, other parameters may change with time, though for the sake of simplicity—and the lack of historical observations to constrain different model components—they remain static in this study.

### 3.2. Evolution of the transport network

The transport network *G* = (*V*, *E*) grows in response to changes in population (figure 1*c*) and the location problem for the creation of new stations is considered here to be stochastic [22]. Specifically, a new station at location *X* is created when, for a random number *Z*(*X*, *t*) ∈ (0, 1], *Q*(*X*, *t*) > *Z*(*x*, *t*) for

3.11a $$Q(X,t)=g\cdot \frac{dP}{dt}\cdot \frac{q(X,t)}{\int_{X}q(X,t)\;dX},$$

where *g* is an exogenous growth factor that ensures *Q*(*X*, *t*) ≤ 1, and *q*(*X*, *t*) is defined as the probability of hosting a new station:

3.11b $$q(X,t)=\frac{\int_{k\in V}\rho (X_{k},t)d_{X,X_{k}}^{-\phi }\;dX}{\int_{k\in V}\;d_{X,X_{k}}^{-\phi }\;dX}+\epsilon l,$$

where *X* is the grid cell to which potential node *k* belongs, and $d_{X,X_{k}}^{-\phi }$ is a spatial kernel allowing the importance of *ρ* to decay with the distance *d* (km) of an area *X* to nearby population in area *X*_*k*_ [22,65]. This is applied across Ω, such that each grid cell has the opportunity to host a new node. Note that equation (3.11*b*) is based on the theory of gravitational models, widely used in urban modelling [16]. However, the approach is applied here to railway stations and modified to account for the presence of existing rail lines *E* (*l* = 1 when *E* is present, zero otherwise—weighted by ϵ), as stations are more likely to be built on existing lines due to lower infrastructure costs. For simplicity, network edges are assumed to be a straight line between two nodes, rather than considering topography or real-life obstacles.

New station nodes *n* then must be connected to the network in order to calculate accessibility/centrality *A*_*v*_ (for equation (3.6)). Nodes *n* are connected in order of their closeness to the core of the network, producing a radial network pattern around the periphery of the city. The core of the network is the area with the highest density of nodes *ρ*_*V*_.

When a new node *n* is created, it is connected to an existing node *o*. Node *o* is chosen based on its distance from *n* and the population density in the same grid cell, such that the location of *o* maximizes $\rho_{V}(o)d_{n,o}^{-\psi }d(n,o)$ (where $d_{n,o}^{-\psi }$ is a spatial kernel for *ρ*_*V*_ influence on *o*). The direct distance *d*(*n*, *o*) is then compared to the lengths of all paths via nearby existing nodes *m*. An edge *E* = {{*n*, *m*}} (rail line) is created to connect *n* to the network via a *Path* to *o* such that

3.12 $$\mathrm{Path}(n,m)=\left\{ \begin{array}{cc} \mathrm{Path}(n,m,\ldots ,o)\; & \text{if}\;d(n,m,\ldots ,o)<\lambda \cdot d(n,o)\;\\ \mathrm{Path}(n,o)\; & \text{otherwise},\; \end{array}\right.$$

where d(n,m,…,o) is the distance between *n* and *o* via *m*. Factor *λ* is chosen to reflect the cost efficiency of adding a new direct rail line between *n* and *o* as opposed to connecting to an existing rail line. New rail lines typically involve higher infrastructure costs, hence it should be the case that *λ* > 1 at least. Note that this is a very simplified approach; in reality many financial, social, and travel time costs are considered during the planning and construction of railway systems [66]. Hence, more complex optimization methods could be considered in the future [67,68].

### 3.3. Simulations setup

#### 3.3.1. London, UK

The first set of model simulations focus on the Greater London area for the period 1831–2011. The domain covers an area of 84 km × 66 km centred on the geographical centre of 2011 Greater London and has a grid resolution of 1 km (figure 1*a*). Three sets of model simulations are included. First, the population density model, i.e. equation (3.1), is solved numerically using the finite difference method and the observed network as model input (results presented in §4.1). The simulation is initialized with the 1831 population data and forced by the growth of the observed rail and underground network (*G* = (*V*, *E*)). The population is estimated at a dt=1 yr time resolution, while the observed network grows at five year time steps starting from 1836. Included stations and rail lines (*V*, *E*) are assumed based on the 2011 network and do not include any stations or lines that have closed prior to this. The network extends beyond the domain for one station according to the 2011 network. As such, the accessibility of well-connected stations in satellite towns might be underestimated as *A*_*v*_ (equation (3.6)) is calculated relative to the network within the domain only (i.e. not nationally). Connections are created between existing nodes when intermediate nodes are not yet present. Results are taken inside a 6 km boundary from the edge of the domain to avoid any boundary effect, as the model uses Dirichlet boundary conditions with *ρ* = 0. A sensitivity analysis is also performed to test the impact of different parameter values and select the best-performing parameterization of equation (3.1) (see electronic supplementary material for details).

In the second set of simulations, the stochastic network model is run to reproduce the response of the railway system to the observed population at a d*t* = 10 yr time resolution (due to the availability of the observed population data) across the same domain (results presented in §4.2). Edges *E* are formed by considering the relative density of the network. New stations are created in response to local population density and presence of existing *E*, such that the evolution of the network aims to optimize the efficiency of population mobility and infrastructure costs. Note that the generated networks differ between runs due to the stochastic nature of the transport model.

Finally, a fully coupled simulation is run to fully explore the coevolution of population and transport network with both systems responding to one another (results presented in §4.3). Initial conditions and parameter values remain the same as for the uncoupled runs (see electronic supplementary material). The transport network is influenced by the population distribution from its initiation in 1841, and grows at a dt=10 yr time resolution to avoid changing time-dependent parameters from the uncoupled run, while the population evolves with dt=1 yr. As with the population density model, the transport network does not affect population growth until the end of phase I.

Note that geographical effects (e.g. topography, rivers etc.) are neglected throughout due to the coarse resolution employed here but they can be easily included as boundary conditions. Further details on the model parameterization can be found in the electronic supplementary material.

#### 3.3.2. Sydney, Australia

To test the generalizability of our approach, we also run model simulations for Metropolitan Sydney, Australia. The Sydney domain covers an area of 84 km × 105 km at a 1 km × 1 km resolution, and simulations are performed for the period 1851–2011. Due to local peculiarities, only the simulated response of the population as forced by the observed rail and tram network is studied for this case (see §4.4). Again the population estimates are at a dt=1 yr time resolution and the observed network grows at 5-year time steps starting from 1856. The presence of the coastline requires mixed boundary conditions. Dirichlet boundary conditions are set on land boundaries, but a mask is used along the coastline such that the population cannot diffuse into the ocean, with the coastline treated as a hard boundary.

## 4. Results

### 4.1. London population

**Figure 2.**
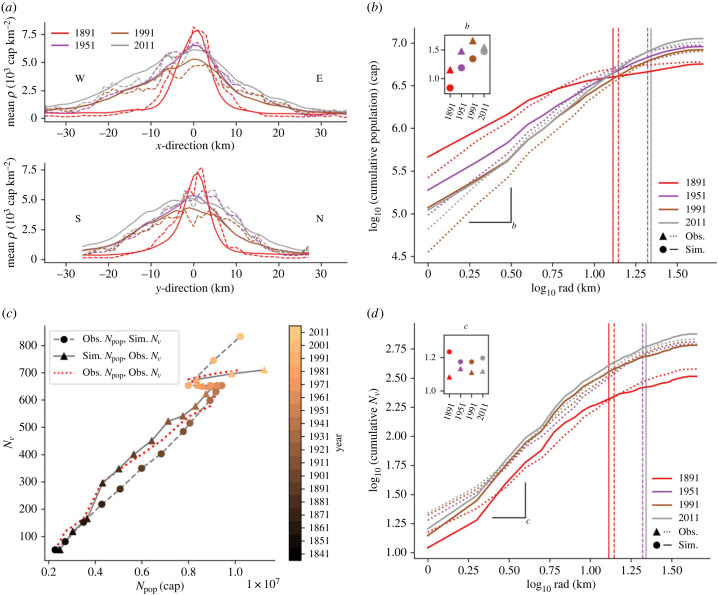
Observed and simulated population profiles. (*a*) Greater London (GL) mean population profiles across the *x*- and *y*-axes of *ρ* (10^3^ cap km^−2^) of observed (dashed) data and simulated (solid, as a response to the growth of the observed rail network) results and (*b*) a log–log scale plot of GL cumulative population (cap) at radius rad (km) from the centre of the domain $\Sigma_{\mathrm{rad}}N_{\mathrm{pop}}$, observed and simulated. Slope *b* is calculated (inset) until an estimated city boundary radius which was found using the City Clustering Algorithm [69]. (*c*) GL total population (*N*_pop_, cap) versus total number of stations (*N*_*v*_) through time. Results for the two feedbacks are shown: observed *N*_pop_ versus simulated *N*_*v*_ (circles and dashed) and simulated *N*_pop_ versus observed *N*_*v*_ (triangles and solid line), along with observed *N*_pop_ versus observed *N*_*v*_ (red dashed line). (*d*) A log–log scale plot of cumulative number of stations at radius rad (km) from the centre of the domain ($\Sigma_{\mathrm{rad}}N_{v}$), observed and simulated. Slope *c* is calculated (inset) until the same radii as (*b*).

Observed and simulated profiles of London population are illustrated in figure 2 for the period 1891–2011. Note that here we focus on population density gradients, thus avoiding the debated issue of defining city boundaries [69–71]. With regards to spatial patterns, studies suggest that the population density of a city follows a power law function of its distance from the city centre [72,73]. This was demonstrated for the case of Greater London’s active population by Li *et al.* [72], but the time evolution of such relations has not previously been explored. Figure 2*b* shows a log–log relation of cumulative *N*_pop_ and radial distance *rad* at different times. A linear relation on the log–log plot with slope *b* is evident from the centre of the city until a limiting distance where it begins to plateau, which could indicate the city’s boundary.

The magnitude of the slope *b* indicates the competition between core and suburban populations—higher *b* implies a greater population towards the suburbs, while lower *b* shows more concentration around the city core. Observed *b* arcs throughout the study period (figure 2*b*, inset). This reveals that the slope is not constant but varies through time, thus encapsulating information on the ‘age’ of a city. Low *b* (1.15) in 1891 reflects the dense population, with the log-linear trend (and hence the city boundary) ceasing at a 14 km radius (*b* is estimated considering data until this point). Suburbanization causes *b* to peak at 1.66 in 1991, after which the population in the centre increases slightly leading up to 2011 (figure 1*d*). Simulated *b* values are lower than observed values for 1891, 1951 and 1991, showing that the model’s population is more concentrated towards the city centre. This is possibly a result of too little *R*_−_ from the city centre (equation (3.7*a*)), which could be improved by adjusting the urban economics parameters seen in equation (3.4*b*).

**Figure 3.**
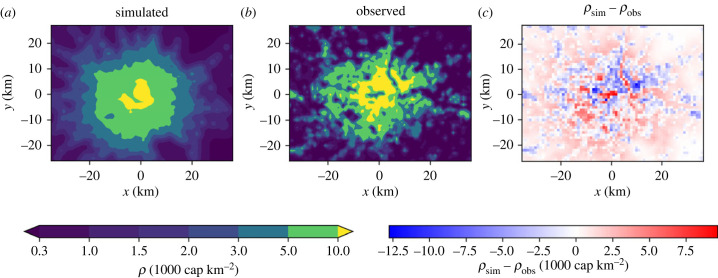
Simulated and observed population density in London (*ρ*) for 2011. (*a*) *ρ*_sim_ as a response to the growth of the observed network; (*b*) *ρ*_obs_; (*c*) *ρ*_sim_ − *ρ*_obs_.

Despite its simplicity, the model agrees well with these observations—small-scale differences occur (figure 3; figure SI.2 in the electronic supplementary material) but the population profiles during the different growth phases and the scaling and evolution of cumulative *ρ* concur (figure 2*a*,*b*; table SI.1 in the electronic supplementary material), confirming the ability of equation (3.1) to reproduce the macroscopic space–time dynamics observed in London (see also electronic supplementary material, figures SI.3, SI.4). Specifically, mean absolute error and root mean squared error for the *x* and *y* profiles of observed and simulated *ρ* (figure 2*a*) are low relative to the overall magnitudes, and *R*^2^ values show a good fit (electronic supplementary material, table SI.1).

Comparisons between observations and model results imply the existence of two distinct phases: a first phase (I, until 1891) of localized diffusion-limited growth, followed by a spatial spreading (II) influenced by the development of the rail network. In general, the spatial spreading of population density is regulated by the factor *η* in equation (3.1) which distributes the new residents across the heterogeneous landscape depending on their net income *Y* (see equations (3.4*a*)–(3.6)). During the initial stage of growth I, job opportunities and wealth creation were promoted in denser urban areas (i.e. *Y* ∼ *ρ* and *R* = *S* = 0) and population concentrated around the city core with a limited exploration of new areas. This is encoded here in the diffusion coefficient and is consistent with historical observations of London’s street intersection density [59]. After this, we see the population responding to the growth of the rail network as travel times over long distances shorten and suburbanization starts to occur (phase II). During the first part of this second phase (II.1), urban sprawl is regulated by a trade-off between living (*C*_*L*_(*X*, *t*)) and transport costs (*C*_*T*_(*X*, *t*)) against the gross income of residents *Y*_gross_, i.e. *Y* = *Y*_gross_ − *C*_*L*_ − *C*_*T*_ [5,57]. Denser urban areas have higher *C*_*L*_ due to higher rent and property values while *C*_*T*_ is directly linked to the network topography [5] and the accessibility of railway stations (see equations (3.5), (3.6)). Areas with lower net income *Y* thus attracted fewer new inhabitants, and areas with negative net income even experienced a population decline (*R*_−_ > 0), promoting relocation to more economically attractive zones (via *R*_+_). During the second part of the network-dominated phase (II.2), additional dynamics are observed. From 1951 to 1991 there is an overall population decline *S*, while suburbanization *R* continues (figure 1*c*,*d*). All areas experience a decline, but those with a lower *Y* experience greater loss, reflecting the repulsive force of lower net income and the resulting migration away from dense housing and inaccessibility. After this, there is a new population increase from 1991 to 2011 (phase II.3) representing a new cycle of innovation and growth [3,55,64].

### 4.2. London rail network

**Figure 4.**
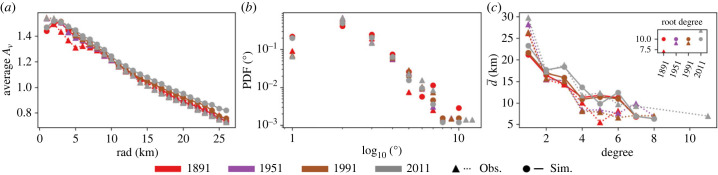
Spatial characteristics of the observed (triangle) and simulated (circle) rail networks for the domain for years 1891, 1951, 1991 and 2011 across the London network, including: (*a*) the average accessibility *A*_*v*_ distance *rad* (kilometre) from centre; (*b*) probability distribution of node degree across the network on log–log scale; (*c*) average distance $\bar{d}$ between the root node of highest degree (inset) and nodes of same and lesser degree [74].

We now turn our attention to the growth of London’s railway system—which evolved in response to the growing urban population (e.g. figure 1*c*). Observations from London reveal a network that grows steadily with the population, branching out from the core city to explore surrounding areas and gradually encouraging urban sprawl (figure 1*a*; figure SI.5 in the electronic supplementary material). Accessibility *A*_*v*_ of a node *v* describes the opportunity of movement across the network as the ratio of total travel distance between all other nodes and the network average travel distance [62]. The spatial variability of *A*_*v*_ remains similar throughout the observation period, indicating that the network is constantly focused on the centre of London (figure 4*a*).

In a similar way to population, we explore the slope *c* of the cumulative number of stations with the radial distance *rad* from the city centre (figure 2*d*). According to theoretical results presented elsewhere [72], there should be a linear relation between the slopes of population, infrastructure and social activities (see figure SI.6 and discussion in electronic supplementary material), hence *c* should increase as the network begins to explore the city’s surroundings. However, this is not the case here as by 1891 the observed value for *c* (1.08) is already at its highest (figure 2*d*). Also, previous work has focused on power law exponents but, strictly speaking, no power law relation was found here. The early stages of network growth, with stations initially created on the city outskirts before the introduction of the underground, therefore still dominate. The 1951 value (1.13) drops below that of 1891, but then grows again in 2011, possibly implying a response of (or response by) the network to the growing suburban population after an initial focus on improving accessibility around the city centre.

It must be noted, however, that *c* is more difficult to interpret than *b*, as the stations are most often fixed in space while the population distribution fluctuates through time. A better comparison would be achieved by comparing total active (residential + workplace) population and the rail network, but observations of working population are not available on such long timescales.

Regarding the properties of the network, in figure 4*c*, the node of the highest degree is chosen (if multiple then the one closest to the centre is selected) and the average Euclidean distances $\bar{d}$ between this node and nodes of lesser degree are shown [74]. For most time steps, $\bar{d}$ decreases as the degree increases, i.e. the nodes of higher degree are more central. This, along with the lower frequency of nodes of higher degree (figure 4*b*), indicates that the network is hierarchical, i.e. the network depends to an extent on transport hubs [75]. The consistency of this pattern over time also suggests that the railway system is self-organizing, a trait seen across many city transport networks and biological organisms (e.g. [43,76–80]).

The time evolution of such network characteristics can be explained by the simple network evolution model presented here (see §3.2). Specifically, we find that the creation of new stations is proportional to the growth rate of the population density (d*P*/d*t*) while the spatial allocation of new stations, which encapsulates planning and decision making, can be approximated by a stochastic process.

For most of the study period, this results in a good agreement between observed and simulated number of stations *N*_*v*_. The simulated transport network follows the growth of the observed population across the run period (figure SI.5 in the electronic supplementary material), mimicking changes in the rate of growth (e.g. during 1911–1931; figure 2*c* and figure SI.5 in the electronic supplementary material). The model does not show the slight increase in stations as seen for the observed network during the population decline (1951–1991), as it is only influenced by population increase (equation (3.11*a*)). Additionally, the network growth responds a bit too sharply to the sudden increase in population after 1991, resulting in a 17% overestimation in *N*_*v*_ at the end of the run (figure 2*c* and figure SI.5 in the electronic supplementary material) and the spatial distribution of stations/railways does not fully capture the observed one. Yet, most network properties are simulated well by the simple model employed here (figure 4). This is in line with our objective of capturing macroscopic characteristics only and points to the need for better descriptions of network formation mechanisms in time and space.

The simulated network also reflects the hierarchical nature of the real network (figures 4*b*,*c*), an important characteristic if we are to reliably quantify and predict the future challenges of urban transport systems. Both networks might display scale-free properties (e.g. [75]), but it is not possible to conclusively determine this from the sample size. In general, there is an ongoing debate as to the prevalence of true scale-free networks in real networks as their growth is influenced by many different processes, making evaluation of finite networks difficult [81–83].

### 4.3. Coevolution of population and transport network

**Figure 5.**
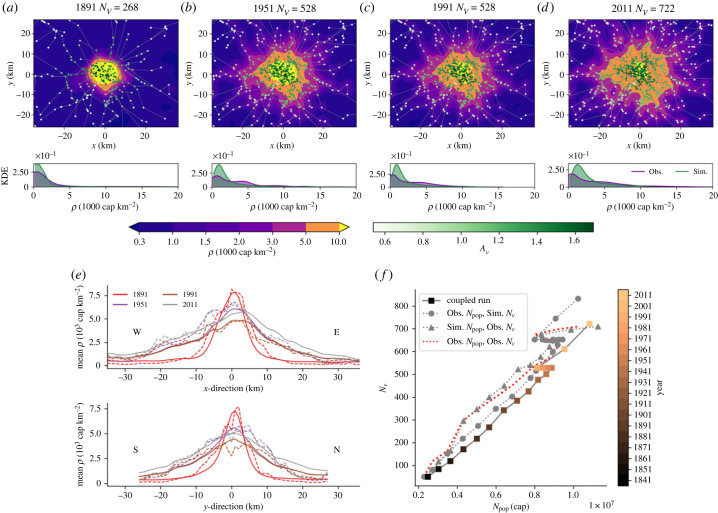
Coevolution of population and railways considering the fully coupled feedbacks, including *ρ* and network (with *A*_*v*_) results. Observed (purple) and coupled run (green) kernel density estimates (KDE) for years (*a*) 1891, (*b*) 1951, (*c*) 1991, (*d*) 2011. Profiles of observed and coupled results for Greater London: (*e*) mean population profiles across the *x*- and *y*-axes of *ρ* (10${\;}^{3}\;{\mathrm{cap}\;\mathrm{km}}^{-2}$) of observed (dashed) data and simulated (solid, coupled run) results for end of phase years 1891, 1951, 1991 and 2011; (*f*) coupled total population (*N*_pop_, cap) versus total number of stations (*N*_*v*_) through time. Results for the two feedbacks in figure 2*c* (see main text) are shown in grey for comparison.

While so far we have analysed the separate growth of population and the railway network, we now focus on their coupled dynamics so that the two systems coevolve and respond to one another—the population grows in response to the transport network and vice versa. For this case, parameter values are kept the same as for the London runs. Despite the simplified approach towards simulating network growth and the focus on ‘first’ principles only (i.e. without any constraints based on local geography or planning), results show both the rail network and population growing in a similar way to the observations, though *N*_*v*_ is slightly lower than the observations and the stand-alone simulation throughout. The city’s shape and the branching patterns of the network are roughly maintained, and statistical features are preserved (figure 5*a*–*e*). The probability distribution of the coupled results shows a higher peak than observations just outside the centre (figure 5*a*–*d*). From early years, simulated stations are more densely located around the centre than observed (figure SI.7 in the electronic supplementary material), leading to higher *C*_*T*_ just outside the centre (approx. 3−7 km) and therefore lower population growth. While the coupled simulation may benefit from refitting of the parameters as opposed to using the same parameters as for the uncoupled runs, such an effort is considered beyond the scope here of testing the model performances. The coupling allows the network to fully display its exploratory abilities as it extends beyond areas of high population—this is crucial for the growth of the city, as subsequent *ρ* distributions spread to congregate around stations. This population spread also agrees well with observations, with *N*_pop_ and *N*_*v*_ following similar trajectories throughout (figure SI.8 in the electronic supplementary material), though there are some differences between observed and simulated kernel density estimate (KDE) distributions towards later years (figure 5).

### 4.4. The case of Metropolitan Sydney

**Figure 6.**
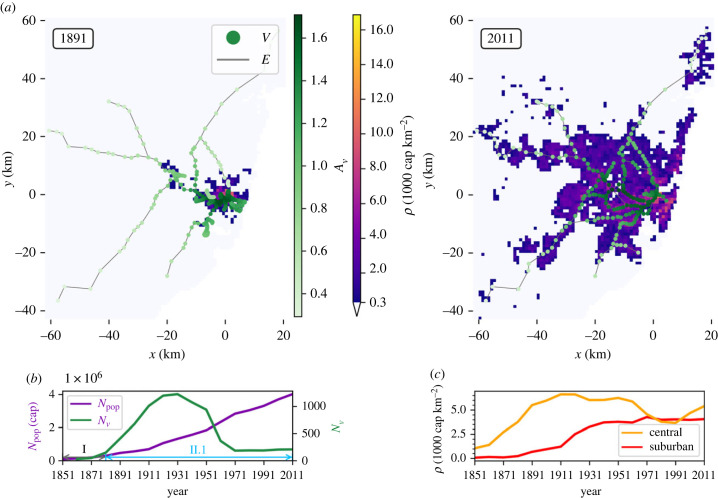
Space–time evolution of Metropolitan Sydney. (*a*) Observed residential population density *ρ* ($1000\;{\mathrm{cap}\;\mathrm{km}}^{-2}$), and rail and tram networks for 1891 and 2011. Maps are centred on the cell containing Sydney Town Hall. Station nodes (*V*) are coloured by accessibility *A*_*v*_. Rail line edges (*E*, grey) are represented by straight lines, which connect stations according to the real network topography but are not representative of the actual geography. For the Sydney model domain, the spatial discretization is d*x* = d*y* = 1 km. (*b*) Total number of rail stations and tram stops (*N*_*v*_, green) and total population (*N*_pop_, cap, purple) within the entire domain for the study period 1851–2011, and the phases of diffusion-limited (grey arrows) and reaction–diffusion (blue arrows) growth. (*c*) *ρ* (cap km^−2^) within representative central (orange) and suburban (red) areas.

The model’s flexibility is now demonstrated through its application to the metropolitan area of Sydney. As a coastal city, Sydney’s population lacks the concentric shape typical of large inland cities such as London and Paris (see figure SI.1 in the electronic supplementary material). The population centre grew slightly inland of Sydney Harbour, and developed along the coast (figure 6*a*), with two large public transport networks dominating the city throughout its history. A rail network has served Sydney since 1855, with a dense network existing in the city centre and inner suburbs, and individual lines reaching small towns north, west and south of the metropolitan area. A large tram network also ran through Sydney’s centre and inner suburbs, starting as a horse-drawn service in 1861 until the closure of the fully electrified network in 1961 in favour of private transport and buses (figure 6*b*). Both networks effectively combine to a single network—the train network is itself fully connected but each tram line has at least one train station a short walking distance from a tram stop. Evidence suggests the existence of a positive feedback between Sydney population and these two rail systems [84], so both networks are considered for this case study.

Unlike London, there is no evidence of a decrease in total population at any point during the study period (figure 6*b*). Hence, only phases I (1851–1881) and II.1 are considered.

In contrast to the London network, the Sydney network is less consistent through time due to the removal of the tram network (figure 6*b*; figures SI.10, SI.11, SI.12 in the electronic supplementary material). The pre-1961 population around the tram network is maintained, with travel dependencies switching from public to private transport. The closure of the tram network altered the dynamics of the entire network, disrupting the linear relation between number of stations and population (electronic supplementary material, figure SI.10) as seen for London. Despite an initial positive feedback between the tram network and population [85], population was found to be more concentrated in train station catchment areas at later dates [86]. The removal of the tram network therefore presents a large-scale intervention that has altered the dynamics of the population growth. Most notably, the removal of the tram network greatly alters the distribution of radial average *A*_*v*_, making the centre of the city less accessible to the rest of the network (figure SI.12*a* in the electronic supplementary material).

**Figure 7.**
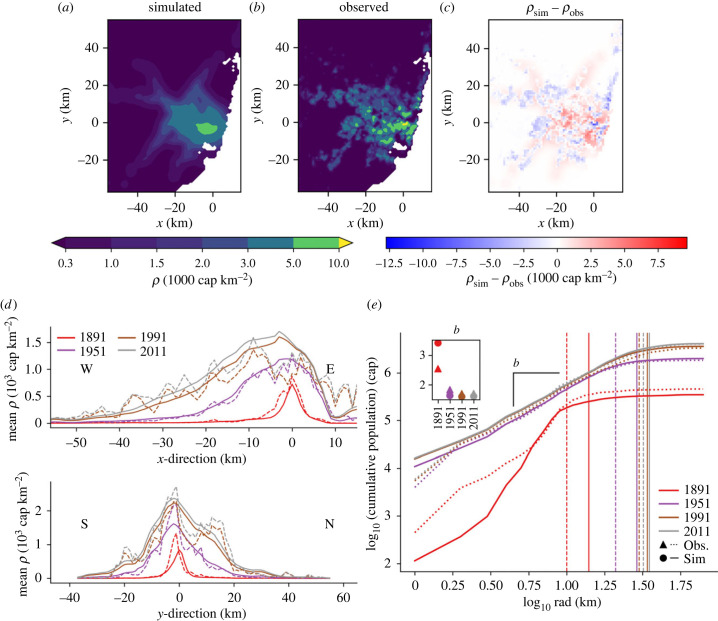
Observed and simulated population for Metropolitan Sydney. (*a*) 2011 *ρ*_sim_ as a response to the observed network. (*b*) 2011 *ρ*_obs_. (*c*) *ρ*_sim_ − *ρ*_sim_. Note that for (*a*–*c*) colour scales are the same as figure 3. (*d*) Sydney mean population profiles across the *x*- and *y*-axes of *ρ* ($10^{3}\;{\mathrm{cap}\;\mathrm{km}}^{-2}$) of observed (dashed) data and simulated (solid, as a response to the growth of the observed rail network) results and (*e*) a log–log scale plot of cumulative population (cap) at radius *rad* (km) from the grid cell containing Sydney Town Hall, observed and simulated. Slope *b* is calculated (inset) until an estimated city boundary radius found using the City Clustering Algorithm [69].

Despite this change in transport modes, simulated population follows the main features of observations, with growth centred on the central Sydney area and gradually spreading to the suburbs (figure 7; figure SI.13 in the electronic supplementary material). Simulated population is successfully maintained in the areas of the city where tram stops were removed after 1961 (figure 6*a*; electronic supplementary material, figure SI.13). Slope *b* of the log-scaled population agrees well for later years figure 7*e*.

There are however a few disparities between simulations and observations. Central *ρ* grows more slowly in response to the network after phase I than observed values (figure 7*b*). *A*_*v*_ is skewed away from the historic centre to favour the western suburbs due to the shape of a network that focuses on branching out rather than central concentration (figure 6*a*, 1891). Simulated profiles also show a much lower *ρ* northeast of the city compared to observations (figure 7; electronic supplementary material, SI.13). The Northern Beaches have always been more remote than other areas at similar distances. The Spit Bridge, over Middle Harbour was not bridged until 1924. Sydney Harbour itself was not bridged (with a rail and tram as well as highway link) in 1932. The model does not account for the alternative attractions (e.g. coastal properties) of living in areas that are poorly served by rail. This highlights the limitations of considering the railway system only, as a multi-modal network that includes road transport may provide more accurate representation of areas that grow independently of the public transport.

## 5. Discussion

Our results from all sets of runs show that the urbanization process can be described by the interplay of two controlling variables: the ‘mass’ of resident population and the urban transport network (approximated by the railway system here) in a similar way to resource allocation by vascular networks. This supports the well-known analogy between cities and biological systems (e.g. [87]). Many urban properties scale with urban population (e.g. [3,10]) and such allometric scaling, originally observed in biological systems [88], is the result of how resources are transported through a branching network (e.g. [89,90]). Over time, the network evolves to better serve the changing population, while the population density varies with the accessibility and attractiveness of different urban areas that depend on a local (rather than global) trade-off between income, transport, and rent costs (e.g. [5,13,57]). These patterns of growth can be seen in cities other than London and Sydney, for example Paris and Washington DC, and for different transport networks (see figure SI.9 in the electronic supplementary material). Similar displays of morphogenesis are observed in biological systems, such as the growth of slime moulds [67] and mycelium [91], whose branching forms self-organize to provide efficient access and delivery of nutrients [80]. In particular, urban dynamics show striking similarities with tumour angiogenesis where a first stage of *in situ*, diffusion-limited growth is followed by neovascularization and development of solid malignant tumours [92]. Such an analogy, which can be applied to urbanization from city to global scales [93], is not new to the scientific (e.g. [41]) and architectural communities (at the 15th Venice Biennale of Architecture, an installation titled ‘urban angiogenesis’ was presented—see [94]). This parallel with cancer raises fundamental questions on the long-term relation between humans and the Earth system [93] but may also provide powerful ideas to transfer knowledge and tools across disciplines (e.g. [41]). For example, policies aimed at regulating the development of road, metro and railway networks may constrain future urban growth patterns in a similar manner as strategies aimed at controlling vascularization and cell-to-cell interactions can mitigate cancer growth (e.g. [41,95]).

For London and Sydney, we have shown that the evolution in the separation between residence and workplace can be described by changes in system dynamics after the advent of the railway in the 1850s. Future changes in mobility and behavioural patterns (e.g. smart-working facilitated by technology and IT infrastructures, post-pandemic changes in workplace occupancy rates, new modes of transport) may generate a new regime shift. How cities will adapt and evolve in response to such changes remains an exciting subject for future enquiry. In general, the framework presented here can be further extended to consider multi-layer transport networks (e.g. roads and rail), daily variations in population density (e.g. [96]), the development of commercial/residential areas and other urban characteristics (e.g. building height [97]), and social segregation of wealthy and poor communities (e.g. [7]). Urban growth modelling itself could also benefit from the increasing availability of high resolution data from rapidly urbanizing countries (e.g. [98]), allowing for better understanding of the modern controls on urban growth dynamics, while in turn providing information for sustainable growth. In general, the model presented in this study would benefit from additional empirical testing. For example, fine-grain data on land use change and residential population could help supporting or refining the assumptions behind the RD dynamics and the sink and redistribution terms employed here.

## 6. Conclusion

We have provided a novel description of urban growth by reformulating classical theories on population dynamics, urban economics and branching transport networks, showing that the space–time evolution of population density in London and Sydney over the past two centuries is regulated by the growth of a hierarchical transport system. Such dynamics can be described by a partial differential equation accounting for exogenous growth, spatial attractiveness and diffusion, coupled with an adaptive network evolution model.

Given the observed similarities among cities (e.g. [73]), the implications of this work are broad and far reaching because a quantitative description of city behaviour can then be employed to predict and optimize future urbanization trajectories. For example, such a reduced-complexity approach is particularly attractive to investigate future urbanization patterns based on transport planning choices as well as to assess the impact of future urban scenarios on various livability indicators (e.g. walkability, pollution, health [35]). This, however, will require the inclusion of city-specific initial and boundary conditions as well as additional processes (e.g. topographic effects, space–time dynamics of passenger and material flows, multi-model transport, energy consumption, urban climate) at the relevant spatio-temporal scales. With such improvements, reduced-order descriptions of the multi-sectoral dynamics occurring in cities will be instrumental for the development of digital twins of the Earth system [99].

Existing top-down urban planning efforts generally fail to recognize cities as a combination of multiple interacting systems [100]. Our results show that, at a macroscopic level, such complex urban dynamics can be described by simple rules and governing equations (e.g. [72]) thus opening up the possibility of exploring future scenarios of change in a more effective and scientifically sound way—a crucial step to address the pressing issues surrounding urban development and its sustainability and health targets [101]. Yet, given the multiple processes and time scales involved, design and decision making should embrace the stochastic nature of urban development and acknowledge the emergence of large-scale patterns that result from the processes occurring at smaller scales.

## Data Availability

All historical and projected residential population data and respective GIS shapefiles were retrieved at the finest possible spatial unit for Greater London (Parish-level, Enumeration District or Output Area) [102–106] and Metropolitan Sydney (Mesh Block) [107]. These were converted from the irregular administrative units to a 1 km × 1 km on the EPSG:27700 (British National Grid) EPSG:8058 (NSW Lambert) coordinate reference systems using spatial overlays via the geopandas (v. 0.9.0) package. The observed data spatial units are coarser for earlier years than for later years. Data used for creating the Greater London observed network are from Levinson [46] and Brown [108], while the Sydney tram [109] and train [85] networks were collated in [84]. These include the location and opening year of stations and the opening year of the connections between them. Node accessibility *A*_*v*_ [62] is calculated during pre-processing. Observed and simulated networks are handled as networkx (v 2.4) Graph objects. All edges *E* are assumed to be bi-directional, and changes between subnetworks or lines (e.g. National Rail, Underground) are assumed to be instant (i.e. no travel time in between). The *V* and *E* within the networkx graph are converted to vector objects then to raster objects for use within the model. All data and code are available upon request to the corresponding author at https://zenodo.org/record/7625769 [110]. Supplementary material is available online [111].
